# Multiparameter MRI-based radiomics analysis for preoperative prediction of type II endometrial cancer

**DOI:** 10.1016/j.heliyon.2024.e32940

**Published:** 2024-06-13

**Authors:** Yingying Cao, Wei Zhang, Xiaorong Wang, Xiaojing Lv, Yaping Zhang, Kai Guo, Shuai Ren, Yuan Li, Zhongqiu Wang, Jingya Chen

**Affiliations:** a Department of Radiology, Affiliated Hospital of Nanjing University of Chinese Medicine, Nanjing, Jiangsu Province, China; b Department of Radiology, Affiliated Hospital of Integrated Traditional Chinese and Western Medicine, Nanjing University of Chinese Medicine, Nanjing, Jiangsu Province, China; c Nanjing University of Chinese Medicine, Nanjing, Jiangsu Province, China; d Taixing People's Hospital, Jiangsu, China; e Department of Radiology, Shandong Provincial Hospital Affiliated to Shandong First Medical University, Jinan, China

**Keywords:** Endometrial carcinoma, Multiparametric magnetic resonance imaging, Radiomics, Nomogram, Machine learning

## Abstract

**Objectives:**

This study aimed to develop and validate a radiomics nomogram based on multiparameter MRI for preoperative differentiation of type II and type I endometrial carcinoma (EC).

**Methods:**

A total of 403 EC patients from two centers were retrospectively recruited (training cohort, 70 %; validation cohort, 30 %). Radiomics features were extracted from T2-weighted imaging, dynamic contrast-enhanced T1-weighted imaging at delayed phase(DCE4), and apparent diffusion coefficient (ADC) maps. Following dimensionality reduction, radiomics models were developed by logistic regression (LR), random forest (RF), bootstrap aggregating (Bagging), support vector machine (SVM), artificial neural network (ANN), and naive bayes (NB) algorithms. The diagnostic performance of each radiomics model was evaluated using the ROC curve. A nomogram was constructed by incorporating the optimal radiomics signatures with significant clinical-radiological features and immunohistochemistry (IHC) markers obtained from preoperative curettage specimens. The diagnostic performance and clinical value of the nomogram were evaluated using ROC curves, calibration curves, and decision curve analysis (DCA).

**Results:**

Among the radiomics models, the NB model, developed from 12 radiomics features derived from ADC and DCE4 sequences, exhibited strong performance in both training and validation sets, with the AUC values of 0.927 and 0.869, respectively. The nomogram, incorporating the radiomics model with significant clinical-radiological features and IHC markers, demonstrated superior performance in both the training (AUC = 0.951) and the validation sets (AUC = 0.915). Additionally, it exhibited excellent calibration and clinical utility.

**Conclusions:**

The radiomics nomogram has great potential to differentiate type II from type I EC, which may be an effective tool to guide clinical decision-making for EC patients.

## Highlights

• Multiparametric magnetic resonance imaging radiomics can be used to predict type II endometrial cancer.
• A nomogram integrated with radiomics signature, clinical characteristics, and immunohistochemistry markers from curettage specimens can improve the predictive performance of type II endometrial cancer.
• This radiomics nomogram can serve as an effective tool for the prediction of type II endometrial cancer.

## 1 Introduction

Endometrial cancer (EC) is a prevalent gynecological malignancy, with global incidence rates persistently rising [1]. Based on the histology and molecular characteristics, ECs are categorized into two distinct types [2]. Type I carcinoma, estrogen-dependent and typically associated with endometrial hyperplasia, accounts for the majority (80%–90 %) of ECs. It includes grades 1 and 2 endometrial carcinomas, which generally have a favorable prognosis. In contrast, type II carcinoma, estrogen-independent and linked to endometrial atrophy, constitutes the remaining 10%–20 % of ECs [[3], [4], [5], [6]]. It encompasses several high-grade, aggressive histological subtypes, which exhibit a high rate of distant metastasis at the time of diagnosis, including grade 3 endometrioid carcinoma, serous adenocarcinoma, clear cell carcinoma, and carcinosarcoma [5,7,8]. Therefore, type II carcinomas typically have a poorer prognosis than type I carcinomas and are associated with over 50 % of the mortality rate among EC patients [9]. For the various histological types of EC, different surgical strategies and postoperative adjuvant therapies significantly differ [1,10]. Thus, distinguishing between type II and type I carcinomas is crucial for identifying the optimal treatment regimen.

Preoperative dilatation and curettage, an invasive diagnostic method, is commonly used to obtain pathological information regarding EC [11,12]. Furthermore, recent studies have shown that magnetic resonance imaging (MRI) has great potential for predicting the histological characteristics of EC, particularly for assessing the depth of myometrial invasion, the status of pelvic lymph nodes, and parametrial extension, which were unobtainable from preoperative curettage pathology [[13], [14], [15]]. A recent study has shown that semiquantitative enhancement parameters on dynamic contrast-enhanced MRI (DCE–MRI) are highly effective for differentiating between type II and type I EC [14]. Consequently, given its noninvasive nature, MRI should be considered a vital complementary tool to preoperative curettage for the evaluation of EC.

Radiomics enables the extraction of quantitative radiological features that can characterize the heterogeneity of tumors, which may enhance the precision of diagnosis, treatment, and prognostication in oncology [16,17]. Additionally, radiomics has been identified as a promising tool for predicting deep myometrial invasion, lymph node status, and risk stratification in endometrial cancer [[18], [19], [20], [21], [22], [23]]. Liu et al. [24] described an MRI-derived radiomics nomogram for differentiating type II from type I EC. Although their approach was based on only ADC images, this previous research has highlighted the significant utility of MRI-based radiomics signatures in tumor assessment [[25], [26], [27]]. Furthermore, machine learning algorithms can potentially enhance the accuracy and reliability of predictive models. Therefore, we propose that machine learning models integrating radiomics features extracted from multiparametric MRI may achieve a more effective prediction of type II EC.

This study aimed to develop and validate a nomogram using diverse machine learning algorithms to discriminate between type II and type I EC preoperatively. The nomogram integrated a multiparametric MRI-derived radiomics signature, IHC markers from preoperative curettage specimens, and radiological characteristics to comprehensively assess the histological features of the tumor.

## 2 Methods

### 2.1 Patients

**Fig. 1:**
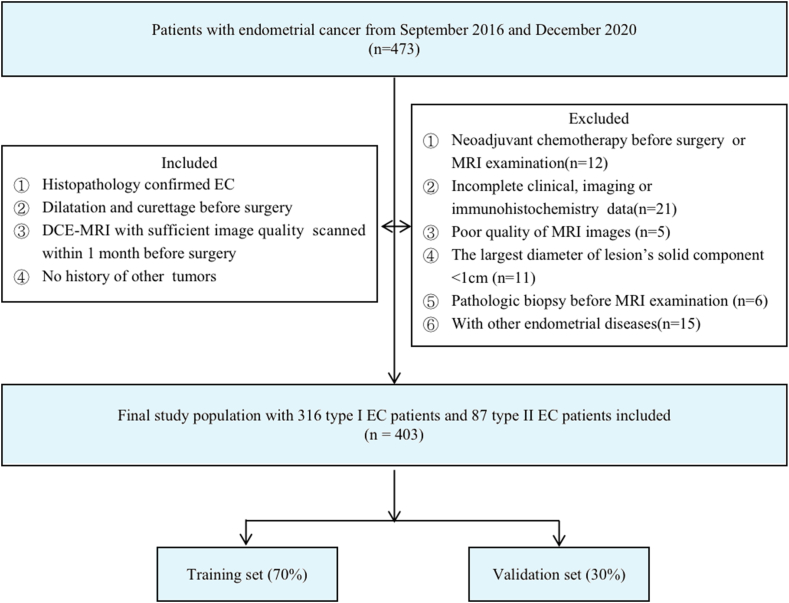
Flowchart of inclusion process of this study.

The ethics committee of the Affiliated Hospital of the Nanjing University of Chinese Medicine and Women's Hospital of Nanjing medical University approved this retrospective study (No.2022NL-KS018), and patient informed consent was waived. This study was conducted in accordance with the Declaration of Helsinki and the Transparent Reporting of a Multivariable Prediction Model for Individual Prognosis or Diagnosis (TRIPOD) guidelines (Supplementary Material 2) [28]. A total of 473 patients with histopathology-proven EC from two Hospitals, enrolled between September 2016 and December 2020, were included in this study. Among these patients, 403 were finally selected for this study based on the following inclusion criteria: (1) histopathology confirmed EC; (2) curettage before surgery; (3) dynamic contrast-enhanced MRI (DCE-MRI) with sufficient image quality scanned within 2 weeks before surgery; (4) no history of other tumors. Notably, 70 patients were excluded based on the following criteria: (1) neoadjuvant chemotherapy before surgery (n = 12); (2) incomplete clinical, imaging, or IHC data (n = 21); (3) poor quality of MRI images (n = 5); (4) the largest diameter of lesion's solid component<1 cm (n = 11); (5) pathologic biopsy before MRI examination (n = 6); (6) with other endometrial diseases (n = 15). Ultimately, 403 EC patients were included and randomly divided into a training and a validation cohort at a 7:3 ratio. A flowchart for patient recruitment is shown in Fig. 1. Baseline clinical data for all patients were retrieved from their medical records, including age, menopause status, and clinical symptoms.

### 2.2 IHC and tissue histopathology

All patients underwent diagnostic curettage before surgery. IHC analysis from curettage specimens, the final histological types, and grades from the surgical specimens were reviewed by a pathologist with 8 years of experience in gynecological malignancies to minimize interpretative errors. The pathologist was blinded to the clinicopathological features of the specimens. For hematoxylin-eosin staining, the curettage samples were processed according to the standard protocol following a 24-h fixation in formalin and embedded in paraffin blocks. For estrogen receptors (ER), progesterone receptors (PR), and Ki-67, IHC results were quantified based on the percentage of positively stained tumor cells. Additionally, *p*53 immunostaining was classified as mutated type (the nuclear staining in >50 % of the tumor cells) and wild type (the nuclear staining in <50 % of the tumor cells). Tumor grade and histological types were also collected based on the final hysterectomy specimens. Endometrioid adenocarcinomas of grades 1 and 2 were classified as Type I EC, whereas Type II EC encompassed grades 3 endometrioid adenocarcinoma, serous adenocarcinoma, clear cell carcinoma, and carcinosarcoma.

### 2.3 MRI protocol

All patients underwent preoperative MR imaging using a 1.5-T scanner (Magnetom Aera, Siemens Healthcare, Erlangen, Germany) covering the whole pelvic area. Imaging sequences included axial T1-weighted imaging (T1WI, time of reception/time of echo(TR/TE), 160/10 ms; section thickness, 5 mm; spacing, 1 mm), axial T2-weighted imaging (T2WI, TR/TE, 1800/80 ms; section thickness, 5 mm; spacing, 1 mm), sagittal fat-suppression T2-weighted imaging (TR/TE, 260/4.6 ms; section thickness, 5 mm; spacing, 1 mm), and sagittal diffusion weighted image (DWI) (TR/TE, 6900/80 ms; section thickness, 5 mm; spacing, 1 mm) with a b-value of 50 and 800 s/mm^2^. Apparent diffusion coefficient (ADC) maps were generated using an automatic post process. Moreover, DCE-MRI was performed at the early arterial phase (DCE1, 15 s), late arterial phase (DCE2, 30 s), parenchyma phase (DCE3, 60 s), and delayed phase (DCE4, 90 s) in the sagittal plane after intravenous injection of 0.1 mmol/kg of Gadolinium diethylene triamine pentaacetic acid (Gd-DTPA).

### 2.4 Conventional MRI data analysis

All MRI datasets were retrieved from the pictures archiving and communication system (PACS). Two pelvic radiologists with 8 and 15 years of MRI experience, respectively, independently analyzed the conventional MRI images. In case of any discrepancies, a consensus was reached after discussion. All radiologists were blinded to the clinical and pathological results. The following qualitative parameters were analyzed: tumor margin, tumor component, myometrial invasion, tumor signal intensity on DWI, tumor signal intensity on T2WI, lymph node status, and abnormal ascites presence/absence. Quantitative parameters included tumor size, ADC value, relative T2 value(r-T2), and enhancement rate of DCE. Definitions of these image features are provided in theSupplementary Material 1.

### 2.5 Image segmentation

**Fig. 2:**
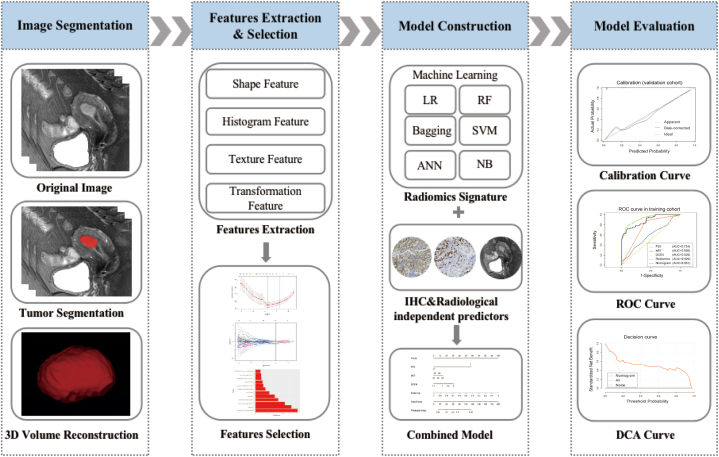
Radiomics workflow.

All images were stored in Digital Imaging and Communication in Medicine(DICOM) format for radiomics analysis. The workflow of radiomics analysis is shown in Fig. 2. Whole volumes of interest (VOI) containing the entire visible tumor were manually segmented independently by two radiologists (with 8 and 15 years of diagnostic experience) using ITK-SNAP software (version 3.2.0, http://www.itksnap.org). ROI for each tumor was delineated layer by layer long tumor margins on the chosen MRI sequences, avoiding adjacent myometrium and endometrium. All radiologists were blinded to the clinical and pathological results. Inter- and intraclass correlation coefficients (ICCs) were employed to assess the reproducibility of segmentation results. One radiologist re-segmented randomly selected MRI images from 30 cases after 3 months, and an ICC value greater than 0.75 indicated substantial agreement.

### 2.6 Radiomics features extraction and selection

All VOIs were processed using AK software (Artificial Intelligence Kit, version 3.0.0, GE Healthcare) for feature extraction. The extracted radiomics features comprised shape, histogram, Haralick, gray-level size zoon matrix (GLSZM) features, gray-level co-occurrence matrix (GLCM), and ray-level run-length matrix (GLRLM). Subsequently, the feature values were normalized and rescaled to the range between 0 and 1.

Following the conventional MRI analysis, ADC and DCE4 sequences were chosen for radiomics analysis. The T2WI sequence was also analyzed as the primary reference for determining the tumor boundaries of other sequences. Therefore, seven feature sets were generated from each patient based on the ADC, T2WI, DCE4, ADC + T2WI, ADC + DCE4, T2WI + DCE4, and ADC + T2WI + DCE4 sequences. Mann-Whitney U tests, Student t tests, and the least absolute shrinkage and selection operator (LASSO) regression model were used to identify the most significant features.

### 2.7 Radiomics signature model construction

We chose six machine learning algorithms to construct radiomics models from each feature set, including logistic regression (LR), random forest (RF), bootstrap aggregating (Bagging), support vector machine (SVM), artificial neural network (ANN), and naive bayes (NB). These models were built using R caret package version 3.7.0, with all parameters retained at their default configurations. A total of 42 models were developed based on six machine learning algorithms and seven feature sets.

### 2.8 Nomogram construction and validation

Significant factors and radiomics signatures were input into a multivariate logistic regression analysis to build a combined model with a nomogram. Odds ratios (ORs) with 95 % confidence intervals (CIs) were calculated for each factor. The nomogram's performance was assessed and validated using the training and validation sets. The Hosmer-Lemeshow test was used to analyze the nomogram's goodness-of-fit. A calibration curve was plotted to assess the consistency between the predicted and actual probabilities. Decision curve analysis (DCA) was conducted to assess the clinical usefulness of the nomogram by quantifying the net benefits at different threshold probabilities.

### 2.9 Statistical analysis

SPSS software (version 21.0, IBM), R software(version 3.7.0, http://www.r-project.org/), and MedCale software (version 19.6.3) were applied for statistical analysis. Continuous variables were compared by performing the Student's t-test or the Mann–Whitney *U* test, while the categorical variables were compared with the chi-squared test or Fisher's exact test. A *P* < 0.05 was considered statistically significant.

## 3 Results

### 3.1 Clinicopathological and IHC characteristics

A total of 403 EC patients (aged 59.1 ± 10.3; 35–78 years) were included in this study. Of these, 316 patients had type I EC, and 87 had type II EC. The clinicopathological and IHC characteristics are summarized in Table 1. No significant differences were observed in menopause status or clinical symptoms between patients with type I and type II EC, except age (p = 0.021). IHC analysis revealed no significant difference in PR expression between the two EC groups; however, ER, Ki67, and p53 expression significantly differed between type I and type II EC(p = 0.032,0.007,<0.001, respectively). Multivariate logistic regression analysis identified ER(OR 0.978; 95%CI 0.957–1.000; p = 0.047), Ki67(OR 1.033; 95%CI 1.010–1.057; p = 0.006), and *p*53(OR 0.123; 95%CI 0.065–0.234; p < 0.001) as independent predictors of type II EC based on clinicopathological and IHC data.

**Table 1:** Patients’ clinicopathological and immunohistochemical characteristics.

Characteristic	Type I (n = 316)	Type II (n = 87)	*p*-value
Age, years a	58 (52–66)	62 (55–68)	0.021^c^
Menopause status^b^			0.558
Postmenopausal status	171 (54.1)	44 (50.6)	
Premenopausal status	145 (45.9)	43 (49.4)	
Clinical symptom^b^			0.85
Abnormal vaginal bleeding	169 (53.5）	53 (60.9）	
Abnormal vaginal discharge	132 (41.8）	42 (48.3）	
Abdominal pain	69 (21.8）	23 (26.4）	
Dyspareunia	65 (20.6）	16 (18.4）	
Asymptomatic	54 (17.1）	13 (14.9）	
Histologic subtype^b^			
Endometrioid, G1	197 (62.3)		
Endometrioid, G2	119 (37.7)		
Endometrioid, G3		46 (52.9)	
Serous adenocarcinoma		21 (24.1)	
Clear cell carcinoma		7 (8.1)	
Carcinosarcoma		13 (14.9)	
ER^a^	50 (35–60）	45 (35–55）	0.032^c^
PR^a^	55 (40–65)	50 (40–65)	0.934
Ki67^a^	40 (33–50）	45 (35–55）	0.007^c^
*p*53^b^			<0.001^c^
Mutated	131 (41.5)	74 (85.1)	
Wild type	185 (58.5)	13 (14.9)	

ER = estrogen receptor; PR = progesterone receptor.

a Median values are reported, with interquartile range in parenthesis.

b Counts are reported, with percentages in parenthesis.

c Statistically significant difference for *p* < 0.05.

### 3.2 Conventional MRI characteristics

Conventional MRI characteristics are summarized in Table 2. No statistically significant differences between the two cohorts were found in tumor margin, tumor component, the signal on DWI, T2WI, ascites, tumor size, r-T2, and DCE. Notably, myometrial invasion, lymph node status, ADC values, and tumor enhancement rate at DCE2, DCE3, and DCE4 were significantly different between type II and type I EC (p = 0.024, 0.011, 0.028, 0.036, 0.032, 0.021, respectively). Multivariate logistic regression analysis identified ADC values(OR 0.101; 95%CI 0.012–0.814; p = 0.031), tumor enhancement rate at DCE4(OR 1.533; 95%CI 1.181–1.989; p = 0.001) and lymph node invasion(OR 0.473; 95%CI 0.249–0.897; p = 0.022) as independent predictors of type II EC in the conventional MRI features.

**Table 2:** Patients’ MR imaging characteristics.

MR features	Type I (n = 316)	Type II (n = 87)	*p*-value
Tumor margin^a^			0.915
Well-defined	253 (80.1）	69 (79.5）	
Ill-defined	63 (19.9）	18 (20.5）	
Tumor component^a^			0.597
Solid	98 (44.9）	34 (39.1）	
Solid-cystic	142 (31.0）	39 (44.8）	
Cystic	76 (24.1）	14 (16.1）	
Myometrial invasion^a^			0.024^c^
Deep	135 (42.7）	49 (56.3)	
Superficial	181 (57.3)	38 (43.7）	
Signal on DWI^a^			0.376
Hyperintensity	293 (92.7)	83 (95.4)	
Isointensity	23 (7.3)	4 (4.6)	
Signal on T2WI^a^			0.382
Heterogeneous	165 (51.3)	40 (46.0)	
Homogeneous	154 (48.7)	47 (54.0)	
Lymph node^a^			0.011^c^
Positive	41 (12.9)	21 (24.1)	
Negative	275 (87.1)	66 (75.9)	
Ascites^a^			0.839
Present	87 (27.5)	23 (26.4)	
Absent	229 (72.7)	64 (73.6)	
Tumor size(cm)^b^	3.64 ± 1.070	3.27 ± 1.424	0.119
ADC ( × 10^−3^ mm^2^/s)^b^	0.781 ± 0.124	0.748 ± 0.110	0.028^c^
r-T2^b^	1.487 ± 0.501	1.540 ± 0.511	0.384
Tumor enhancement rate^b^			
DCE1	0.782 ± 0.196	0.766 ± 0.194	0.492
DCE2	0.819 ± 0.109	0.773 ± 0.128	0.036^c^
DCE3	0.898 ± 0.133	0.960 ± 0.144	0.032^c^
DCE4	0.862 ± 0.198	0.920 ± 0.185	0.021^c^

MRI = magnetic resonance; DWI = diffusion-weighted imaging; T2WI = T2-weighted imaging; ADC = apparent diffusion coefficient; r-T2 = relative T2 value; DCE1, DCE2, DCE3, DCE4 = enhancement rate of dynamic enhancement in early arterial phase, late arterial phase, parenchymal phase, and delayed phase.

a Counts are reported, with percentages in parenthesis.

b Counts are presented, with mean ± standard deviation.

c Statistically significant difference for *p* < 0.05.

### 3.3 Radiomics features selection from multi-sequence

These sequences were further analyzed as the tumor signals on ADC and DCE4 were significantly different between type I and type II EC (p < 0.05). Additionally, T2WI was also interpreted as the reference sequence. 720 radiomics features were extracted from each of the three sequences, demonstrating satisfactory intraobserver reproducibility (ICC = 0.854) and interobserver reproducibility (ICC = 0.832). LASSO analysis of the 7 feature sets (ADC, DCE4, T2WI, ADC + T2WI, ADC + DCE4, T2WI + DCE4, ADC + DCE4+T2WI) finally selected 25 radiomics features for ADC, 7 for T2WI, 40 for DCE4, 14 for ADC + T2WI, 12 for ADC + DCE4, 31 for T2WI + DCE4, and 19 for T2WI + DCE4+T2WI (Fig. S1).

### 3.4 Radiomics signatures construction

**Fig. 3:**
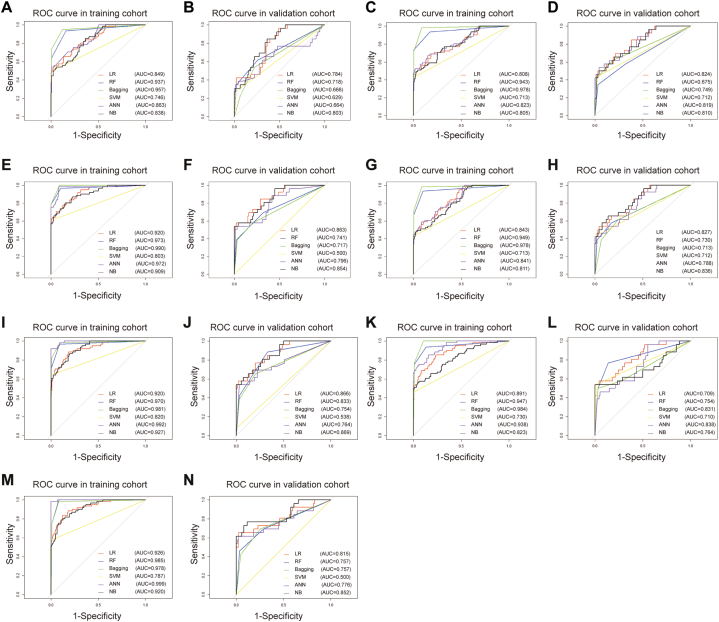
ROC curves of radiomics features using six machine learning algorithms for ADC sequence in the training set(A) and validation set(B); T2WI sequence in the training set(C)and validation set(D); DCE4 sequence in the training set (E) and validation set (F); ADC + T2WI sequences in the training set (G) and validation set (H); ADC + DCE4 sequences in the training set (I) and validation set (J); T2WI + DCE4 sequences in the training set (K) and validation set (L); ADC + T2WI + DCE4 sequences in the training set (M) and validation set (N); AUC, area under the curve; ROC, receiver operating characteristic; T2WI, T2-weighted imaging; ADC, apparent diffusion coefficient; DCE4, enhancement rate of dynamic enhancement in delayed phase. LR, logistic regression; RF, random forest; Bagging, bootstrap aggregating; SVM, support vector machine; ANN, artificial neural network; NB, naive bayesian.

Seven feature sets were selected and entered into six machine learning algorithms (LR, RF, Bagging, SVM, ANN, NB) to construct radiomics signatures, respectively. The ROC analysis results for all classifiers in training and validation cohorts are shown in Fig. 3(A - N). Of the 42 models tested, two NB classifiers utilizing ADC + DCE4 and ADC + DCE4+T2WI features demonstrated superior performance to other classifiers for type II EC. Notably, the NB classifier using ADC + DCE4 features showed higher predictive accuracy than the NB classifier with ADC + DCE4+T2WI features in the validation cohorts (AUC = 0.869 vs 0.852; p = 0.035). Therefore, the NB classifier, incorporating 12 radiomics features from ADC + DCE4, was selected to develop the final radiomics model. This radiomics model performed well in both the training and validation sets, with AUC values of 0.927 and 0.869, sensitivities of 0.869 and 0.615, and specificity of 0.802 and 0.926, respectively, as shown in Table S1. The Rad-score calculation was shown in the following:

Rad−score(ADC + DCE4) = −1.022040822 + 0.03594094 × HaralickCorrelation_angle135_offset4+0.03938213 × Correlation_angle0_offset7+0.04194874 × ClusterShade_angle135_offset7+0.05046682 × histogramEnergy+0.0548972 × ClusterShade_AllDirection_offset7+0.07133994 × ClusterShade_angle90_offset7+0.13946861 × Quantile0.975 + 0.09971917 × Percentile90 + 0.16356268 × ClusterShade_angle0_offset7+0.21334371 × ClusterShade_angle0_offset4+0.26044351 × Variance+0.30610114 × ClusterShade_angle90_offset4.

### 3.5 Radiomics nomogram construction and validation

**Fig. 4:**
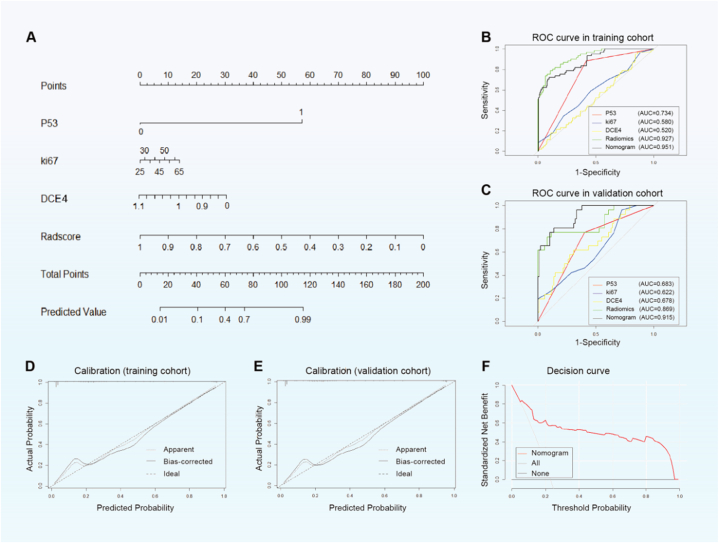
The nomogram development and validation. **A** The nomogram integrating the radiomics model, p53, ki67, and DCE4 was constructed. **B** The ROC curves of the p53, ki67, DCE4, radiomics score, and combined nomogram in the training set. **C** The ROC curves of the p53, ki67, DCE4, radiomics score, and combined nomogram in the validation set. **D** The calibration curve of nomogram in the training set. **E** The calibration curve of nomogram in the validation set. **F** Decision curve analysis for the nomogram. DCE4, enhancement rate of dynamic enhancement in delayed phase; AUC, area under the curve; ROC, receiver operating characteristic.

*p*53, Ki67, tumor enhancement rate at DCE4, and 12 radiomics features from ADC&DCE4 were identified using multivariate logistic regression analysis and chosen as independent predictors to create a visual nomogram (Fig. 4A). The diagnostic performance of each predictor in the training and validation sets was shown in Fig. 4B and C. The combined nomogram yielded higher AUC value than those of the *p*53, Ki67, DCE4, and radiomics model in the training set (AUC = 0.951 vs 0.734, 0.580, 0.520, 0.927) and the validation set (AUC = 0.915 vs 0.683, 0.622, 0.678, 0.869)(Table 3). The calibration curves showed that the predicted probabilities of the nomogram were closely aligned with the actual type II EC estimates in both the training and validation cohorts(Fig. 4D and E). DCA indicated that the combined nomogram was clinically beneficial, showing a high net benefit across a wide range of threshold levels(Fig. 4F).

**Table 3:** The predictive performance of the models.

Variables	Training set			Validation set		
	AUC(95%CI)	SEN(%)	SPE(%)	AUC(95%CI)	SEN(%)	SPE(%)
P53	0.734(0.682–0.787)	88.5	58.3	0.683(0.586–0.781)	76.9	59.8
Ki67	0.580(0.497–0.663)	59.0	54.4	0.622(0.q500–0.743)	96.2	27.6
DCE4	0.520(0.438–0.601)	95.1	15.7	0.678(0.560–0.796)	61.5	69.0
Radiomics	0.927(0.894–0.960）	86.9	80.2	0.869(0.793–0.945)	61.5	92.6
Nomogram	0.951(0.831–0.929）	72.1	90.2	0.915(0.857–0.972)	80.8	86.2

AUC = area under the curve; CI = confidence interval; SEN = sensitivity; SPE = specificity; DCE4 = enhancement rate of dynamic enhancement in delayed phase.

## 4 Discussion

Our study developed a multiparametric MRI-based radiomics model for differentiating type II from type I EC patients. Additionally, the integrated nomogram, which merged radiomics signatures with conventional imaging features and IHC markers from curettage specimens, demonstrated enhanced predictive performance. This model can potentially serve as an effective tool for predicting histological types and aiding clinical decision-making in EC patients before surgery.

Previous studies [29,30] have indicated that reduced expression of ER and PR, elevated expression of Ki67, and P53 mutation are significantly associated with non-endometrioid histology and poor differentiation in endometrial carcinoma. The current study identified elevated Ki67 expression and p53 mutations as independent predictors of type II EC, consistent with previous findings. Reduced ER expression is associated with type II EC, however, this association was attenuated after adjustment for conventional MRI features. Additionally, PR expression did not exhibit a significant association with type II EC, which contradicted previous findings and could be attributed to sample size limitations or the methodology used for scoring immunohistochemical results.

MRI provides high tissue contrast resolution and reproducibility, potentially supplementing preoperative dilatation and curettage with valuable information. Our study found that type II tumors exhibited a significantly higher enhancement rate on DCE-MRI than type I tumors. Few studies have investigated the correlation between contrast-enhanced MRI findings and histology types in EC. Fukunaga et al. [14] found that type II carcinomas demonstrated a greater enhancement degree than type I carcinomas, corroborating our findings. In contrast, Ippolito et al. [31] observed significantly higher DCE-MRI perfusion parameters in G1 compared to G2/3 endometrial carcinomas. The discrepant findings may be attributed to differences in the histological subtypes of EC in that study. Non-endometrioid histology types were excluded from their research, yet they represented 10.17 % (41/403) of all our cases.

In the current study, a radiomics signature derived from combined ADC and DCE-MRI sequences was selected, and the Naive Bayes algorithm was employed to construct the final radiomics model. The association between ADC based-radiomic features and histological subtypes of EC has also been reported in the previous study [24], indicating the discriminative power of ADC maps in identifying increased cellular heterogeneity within high-grade endometrial tumors. Fasmer et al. [32] demonstrated that radiomics signatures derived from DCE-MRI can facilitate preoperative risk stratification for the prediction of aggressive EC, including non-endometrioid histology and high-grade tumors. As in the current study, previous researches [21,33] have also revealed that radiomics signatures derived from ADC and DCE-MRI sequences can detect lymphovascular space invasion in endometrial carcinoma, highlighting the reproducibility of radiomics signatures in representing the aggressive histopathological characteristics of EC.

Furthermore, we developed a predictive model that integrated radiomics signatures with key imaging features and IHC markers, demonstrating improved diagnostic performance compared to risk factors alone. The latest study by Liu et al. [24] developed an ADC-based radiomics nomogram that incorporates radiomics signatures, clinical risk factors, and dilatation and curettage results to predict type II EC, with AUC values of 0.93 and 0.91 in the training and validation set, respectively. However, this was a single-center study, and the model's performance was not validated on an external cohort. Additionally, the authors used only a single ADC map for radiomics feature extraction, overlooking the potential for multiparameter features to provide more comprehensive tumor information.

Radiomics is increasingly utilized to assess EC to evaluate key prognostic factors, including the depth of myometrial invasion, histological grade, lymphovascular space invasion status, lymph node metastasis, and molecular characteristics [34,35]. In addition to radiomics, many other omics methods are used to guide risk stratification and assess the prognosis of gynecologic diseases, such as genomics, transcriptomics, proteomics, and metabolomics [36]. Apart from omics models, various clinical nomograms have also been developed to predict disease progression [37]. A previous study developed a model to predict the probability of pelvic inflammatory disease progressing to sepsis, including dialysis, reduced platelet counts, history of pneumonia, medication of glucocorticoids, and increased leukocyte counts [38]. It's worth noting that radiomics represents a non-invasive and low-cost tool that delivers comprehensive information about lesions, including their morphological relationship with adjacent tissues, which cannot be provided by previously mentioned methods.

In contrast to previous studies, the current multicenter study employed MRI multimodal sequences, providing a more comprehensive radiomics extraction compared to the single-sequence utilized in earlier research. Moreover, despite being the optimal method for histological characterization of EC in current clinical practice, dilatation and curettage results are limited by erroneous sampling locations or inadequate sample amounts [39]. Distinct from prior studies, the predictive model developed in this study integrated microscopic IHC markers from preoperative curettage specimens with macroscopic conventional MRI imaging, potentially enhancing the diagnostic accuracy of preoperative predictions based on dilatation and curettage results. Consequently, this nomogram has the potential to serve as a reliable tool for preoperative prediction of type II EC, aiding in the formulation of early and personalized treatment strategies for patients in future clinical work.

Several limitations of this study should also be noted. First, deep learning is a promising approach that can automatically learn feature representations from images according to clinical objectives and has been widely used in oncological research [40]. However, the number of participants included in this study is limited, especially for patients with type II EC, which may lead to overfitting and poor generalization capabilities of the model. A comparative analysis between machine learning and deep learning models would be a valuable contribution to future research with a larger sample size. Second, automated ROI delineation can be applied to reduce variability in future work. Third, our study did not include IHC markers like p16, WT-1, or other important indicators due to its retrospective, multicenter nature. In the future, we will consider more valuable IHC markers to construct a more comprehensive model for predicting type II EC.

## 5 Conclusion

Our study established and validated an NB model integrating radiomics signatures from ADC + DCE4, radiological characteristics, and IHC markers from curettage specimens to predict the histological subtypes of EC preoperatively. The nomogram, based on this model, exhibited promising performance in both the training and validation sets, potentially serving as an effective guide for preoperative clinical decision-making in EC patients.

## Funding

This study was funded by the 10.13039/501100001809National Natural Science Foundation of China (Grant No. 82171925), and the Innovation and Development Fund of Jiangsu Hospital of TCM (Grant No. y2023cx28).

## Ethical approval

This study was approved by ethical committee of the affiliated hospital of Nanjing university of Chinese medicine (No.2022NL-KS018). Informed consent was waived for this retrospective observational study.

## Inclusion and diversity

We support inclusive, diverse, and equitable conduct of research.

## Data availability statement

Because the information comes from the hospital case system, the data sets generated and analyzed during the current study period are not publicly available, but can be obtained from the corresponding authors on reasonable request.

## CRediT authorship contribution statement

**Yingying Cao:** Writing – original draft, Data curation. **Wei Zhang:** Investigation, Conceptualization. **Xiaorong Wang:** Data curation. **Xiaojing Lv:** Software, Resources. **Yaping Zhang:** Validation, Software. **Kai Guo:** Writing – review & editing, Formal analysis. **Shuai Ren:** Data curation. **Yuan Li:** Methodology. **Zhongqiu Wang:** Writing – review & editing, Conceptualization. **Jingya Chen:** Supervision, Conceptualization.

## Declaration of competing interest

The authors declare that they have no known competing financial interests or personal relationships that could have appeared to influence the work reported in this paper.

This study was approved by ethical committee of the affiliated hospital of Nanjing university of Chinese medicine (No.2022NL-KS018). Informed consent was waived for this retrospective observational study.
