# Supplementary material for: Multiparameter MRI-based radiomics analysis for preoperative prediction of type II endometrial cancer
Source: Heliyon. 2024 Jun 13;10(12):e32940. doi: 10.1016/j.heliyon.2024.e32940 (PMC11234004; doi:10.1016/j.heliyon.2024.e32940)
Supplement: Multimedia component 1 [file mmc1.docx]

**Supplementary Material**

**Conventional MRI data analysis**

As qualitative assessments, the radiologists assessed the following image features of EC: (a) tumor margin, categorized as well-defined margin (80-100% smooth tumor margin) and ill-defined margin(20-100% non-smooth tumor margin); (b) tumor component, defined as solid (≤30% cystic component), solid-cystic(30-60% cystic component), and cystic(60-100% cystic component); (c) myometrial invasion, classified as deep myometrial invasion (≥50% tumor infiltration of the thickness of the myometrium) and superficial myometrial invasion(<50% tumor infiltration of the thickness of the myometrium); (d) tumor signal intensity on DWI, presenting as hyperintensity(more than the adjacent myometrium) and isointensity (similar to the adjacent myometrium); (e) tumor signal intensity on T2WI, presenting as heterogeneous and homogeneous; (f) the status of lymph node, categorized as positive and negative, positive lymph node was defined as following criteria[1]: pelvic lymph node >8mm, or with non-homogeneous enhancement and central necrosis, or with an irregular shape; (g) the presence or absence of abnormal ascites.

As quantitative assessments, the radiologists assessed the following image features of EC: (a) tumor size, defined as the maximum diameter of the tumor; (b) ADC value, defined as the signal intensity of the tumor on ADC maps by placing the regions of interest (ROI); (c) relative T2 value(r-T2), calculated by dividing the tumor signal intensity by that of the myometrium on T2WI; (d) the enhancement rate of DCE images, calculated by dividing the tumor signal intensity by that of the myometrium on DCE1, DCE2, DCE3, and DCE4 maps, respectively. All ROIs were identified manually on the slice of the tumor which exhibited the largest diameter whereas cystic and hemorrhagic areas were avoided.

[1] Yan B, Li Y, Ma F, et al (2021) Radiologists with MRI-based radiomics aids to predict the pelvic lymph node metastasis in endometrial cancer: a multicenter study. Eur Radiol 31:411-422.

**
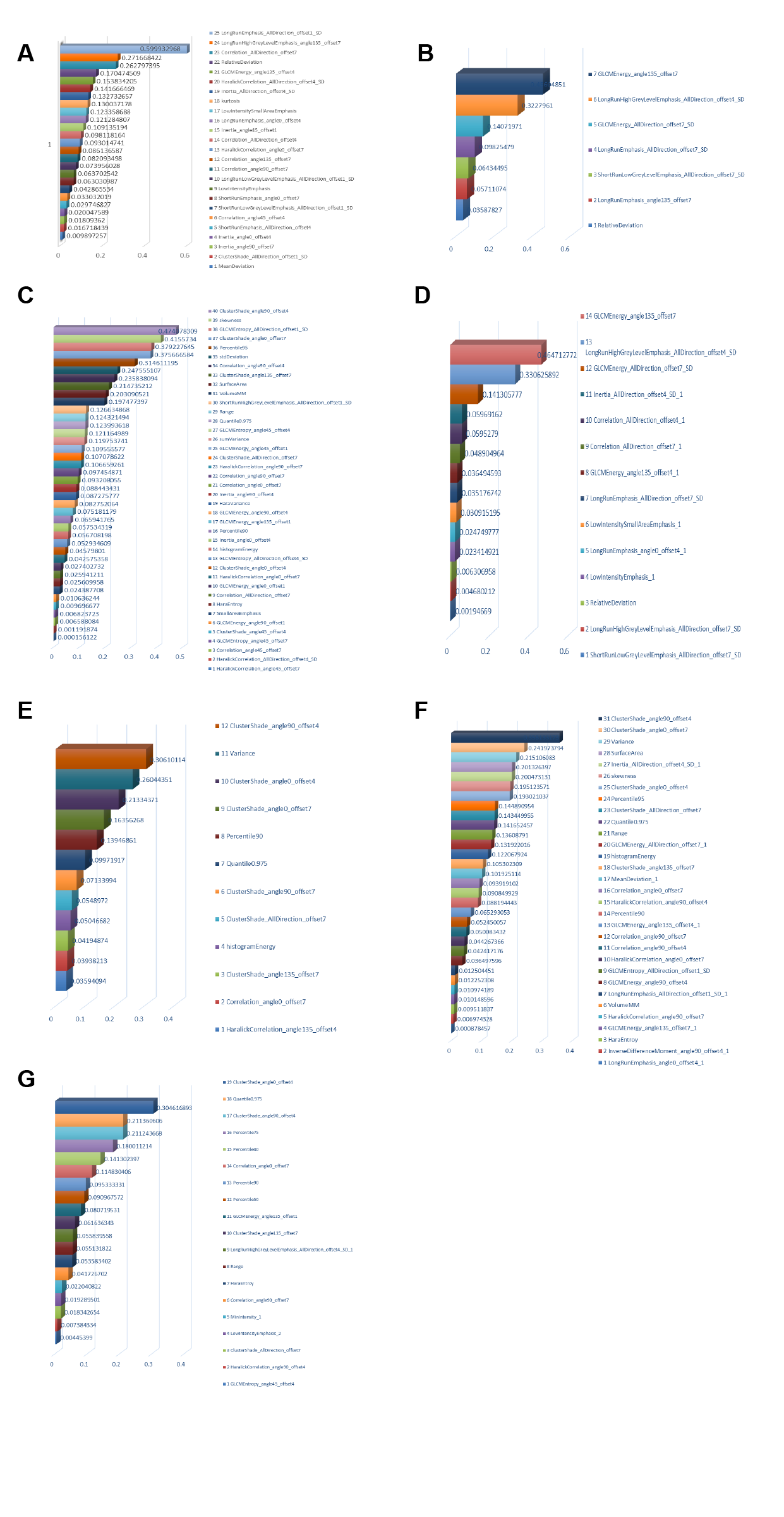
**

**Figure S1.** Radiomic features that retained for model construction. **A** A total of 25 radiomics features for ADC. **B** A total of 7 radiomics features for T2WI. **C** A total of 40 radiomics features for DCE4. **D** A total of 14 radiomics features for ADC&T2WI. **E** A total of 12 radiomics features for ADC&DCE4. **F** A total of 31 radiomics features for T2WI&DCE4.

**G** A total of 19 radiomics features for ADC&T2WI&DCE4. T2WI, T2-weighted imaging; ADC, apparent diffusion coefficient; DCE4, dynamic enhancement delayed phase.

**Table S1 The predictive performance of six machine learning algorithms in the training and test set.**

| Sequences | Algorithm | Training set | | |  | Validation set | | |
| --- | --- | --- | --- | --- | --- | --- | --- | --- |
|  |  | AUC(95%CI) | SEN(%) | SPE(%) |  | AUC(95%CI) | SEN(%) | SPE(%) |
| ADC | LR | 0.849(0.793-0.904) | 65.6 | 86.5 |  | 0.784(0.692-0.876) | 96.2 | 51.1 |
|  | RF | 0.937(0.900-0.974) | 93.4 | 84.2 |  | 0.718(0.604-0.831) | 61.5 | 74.5 |
|  | Bagging | 0.957(0.926-0.989) | 95.1 | 87.8 |  | 0.666(0.556-0.776) | 53.8 | 77.7 |
|  | SVM | 0.746(0.683-0.809) | 49.2 | 100.0 |  | 0.629(0.542-0.717) | 26.9 | 98.9 |
|  | ANN | 0.863(0.812-0.914) | 54.1 | 99.5 |  | 0.664(0.523-0.806) | 38.5 | 95.7 |
|  |  | 0.838(0.784-0.892) | 85.2 | 63.5 |  | 0.803(0.718-0.888) | 84.6 | 63.8 |
| T2WI | LR | 0.808(0.743-0.874) | 68.9 | 81.5 |  | 0.824(0.734-0.914) | 69.2 | 78.7 |
|  | RF | 0.943(0.905-0.980) | 93.4 | 82.9 |  | 0.675(0.555-0.795) | 34.6 | 96.8 |
|  | Bagging | 0.978(0.959-0.997) | 98.4 | 91.4 |  | 0.749(0.64-0.858) | 61.5 | 81.9 |
|  | SVM | 0.713(0.651-0.776) | 42.6 | 100.0 |  | 0.712(0.615-0.808) | 42.3 | 100.0 |
|  | ANN | 0.823(0.760-0.887) | 67.2 | 85.1 |  | 0.819(0.726-0.912) | 53.8 | 95.7 |
|  | NB | 0.805(0.741-0.869) | 49.2 | 97.3 |  | 0.810(0.716-0.903) | 50.0 | 95.7 |
| DCE4 | LR | 0.920(0.882-0.958) | 70.5 | 94.6 |  | 0.863(0.783-0.942) | 84.6 | 71.3 |
|  | RF | 0.973(0.947-0.998) | 96.7 | 89.6 |  | 0.741(0.628-0.853) | 69.2 | 68.1 |
|  | Bagging | 0.990(0.984-0.997) | 100.0 | 91.0 |  | 0.717(0.605-0.829) | 57.7 | 79.8 |
|  | SVM | 0.803(0.741-0.865) | 60.7 | 100.0 |  | 0.500(0.500-0.500) | 100.0 | 100.0 |
|  | ANN | 0.972(0.944-1.000) | 98.4 | 91.9 |  | 0.796(0.694-0.898) | 53.8 | 96.8 |
|  | NB | 0.909(0.867-0.952) | 80.3 | 85.1 |  | 0.854(0.774-0.934) | 53.8 | 100.0 |
| ADC+T2WI | LR | 0.843(0.790-0.896) | 59.0 | 89.2 |  | 0.827(0.790-0.896) | 53.8 | 95.7 |
|  | RF | 0.949(0.914-0.984) | 93.4 | 90.1 |  | 0.730(0.914-0.984) | 57.7 | 83.0 |
|  | Bagging | 0.978(0.959-0.997) | 98.4 | 91.4 |  | 0.713(0.959-0.997) | 61.5 | 75.5 |
|  | SVM | 0.713(0.651-0.776) | 42.6 | 100.0 |  | 0.712(0.651-0.776) | 42.3 | 100.0 |
|  | ANN | 0.841(0.786-0.896) | 59.0 | 91.4 |  | 0.788(0.786-0.896) | 42.3 | 100.0 |
|  | NB | 0.811(0.752-0.871) | 49.2 | 96.4 |  | 0.836(0.752-0.871) | 65.4 | 86.2 |
| ADC+DCE4 | LR | 0.920(0.881-0.959) | 88.5 | 81.5 |  | 0.866(0.788-0.944) | 76.9 | 79.8 |
|  | RF | 0.970(0.945-0.995) | 96.7 | 91.0 |  | 0.833(0.749-0.917) | 88.5 | 67.0 |
|  | Bagging | 0.981(0.926-1.000) | 98.4 | 90.5 |  | 0.754(0.647-0.862) | 65.4 | 79.8 |
|  | SVM | 0.820(0.759-0.880) | 63.9 | 100.0 |  | 0.538(0.486-0.591) | 7.7 | 100.0 |
|  | ANN | 0.992(0.985-1.000) | 91.8 | 100.0 |  | 0.764(0.645-0.882) | 53.8 | 96.8 |
|  | NB | 0.927(0.894-0.960) | 86.9 | 80.2 |  | 0.869(0.793-0.945) | 61.5 | 92.6 |
| T2WI+DCE4 | LR | 0.891(0.843-0.983) | 85.2 | 76.6 |  | 0.709(0.738-0.923) | 53.8 | 100.0 |
|  | RF | 0.947(0.911-0.983) | 93.4 | 86.9 |  | 0.754(0.746-0.930) | 96.2 | 44.7 |
|  | Bagging | 0.984(0.973-0.994) | 100.0 | 89.6 |  | 0.831(0.589-0.831) | 57.7 | 95.7 |
|  | SVM | 0.730(0.666-0.793) | 45.9 | 100.0 |  | 0.710(0.666-0.862) | 50.0 | 96.8 |
|  | ANN | 0.938(0.907-0.969) | 73.8 | 95.9 |  | 0.838(0.649-0.858) | 76.9 | 86.2 |
|  | NB | 0.823(0.759-0.887) | 55.7 | 95.9 |  | 0.764(0.571-0.848) | 53.8 | 98.9 |
| ADC+T2WI+DCE4 | LR | 0.926(0.888-0.964) | 83.6 | 87.7 |  | 0.815(0.703-0.927) | 65.4 | 97.7 |
|  | RF | 0.985(0.974-0.995) | 100.0 | 91.7 |  | 0.757(0.645-0.869) | 46.2 | 96.6 |
|  | Bagging | 0.978(0.958-0.998) | 98.4 | 92.6 |  | 0.757(0.649-0.865) | 69.2 | 74.7 |
|  | SVM | 0.787(0.724-0.849) | 57.4 | 100.0 |  | 0.500(0.500-0.500) | 100.0 | 0.0 |
|  | ANN | 0.999(0.997-1.000) | 98.4 | 100.0 |  | 0.776(0.649-0.903) | 61.5 | 97.7 |
|  | NB | 0.920(0.883-0.957) | 78.7 | 89.2 |  | 0.852(0.753-0.951) | 76.9 | 88.5 |

LR, logistic regression; RF, random forest; Bagging, bootstrap aggregating; SVM, support vector machine; ANN, artificial neural network; NB, naive bayes;

ADC, apparent diffusion coefficient; T2WI, T2-weighted imaging; DCE4, dynamic enhancement delayed phase; AUC, area under the curve; CI, confidence interval; SEN, sensitivity; SPE, specificity.
